# Matrix metalloproteinases and tissue damage in HIV-tuberculosis immune reconstitution inflammatory syndrome

**DOI:** 10.1002/eji.201343593

**Published:** 2013-10-30

**Authors:** Rebecca Tadokera, Graeme A Meintjes, Katalin A Wilkinson, Keira H Skolimowska, Naomi Walker, Jon S Friedland, Gary Maartens, Paul T G Elkington, Robert J Wilkinson

**Affiliations:** 1 Clinical Infectious Diseases Research Initiative, Institute of Infectious Diseases and Molecular Medicine, Faculty of Health Sciences, University of Cape TownCape Town, South Africa; 2 Infectious Diseases Unit, GF Jooste HospitalManenberg, South Africa; 3 Department of Medicine, Imperial College LondonLondon, UK; 4 MRC National Institute for Medical ResearchLondon, UK; 5 Division of Clinical Pharmacology, Department of Medicine, University of Cape TownCape Town, South Africa; 6 Faculty of Medicine, University of Southampton, Southampton General HospitalSouthampton, UK

**Keywords:** Drug therapy complications, HIV-1 infection, Immune reconstitution inflammatory syndrome, Matrix metalloproteinase, Tuberculosis

## Abstract

The HIV-TB-associated immune reconstitution inflammatory syndrome (TB-IRIS) can complicate combined treatments for HIV-1 and TB. Little is known about tissue damage in TB-IRIS. Matrix metalloproteinases (MMPs) degrade components of the extracellular matrix and consequently may play a role in such immunopathology. Here we investigated the involvement of MMPs in TB-IRIS. We determined MMP transcript abundance and secreted protein in *Mycobacterium tuberculosis* stimulated PBMCs from 22 TB-IRIS patients and 22 non-IRIS controls. We also measured MMP protein levels in corresponding serum and the effect of prednisone — which reduces the duration of symptoms in IRIS patients — or placebo treatment on MMP transcript and circulating MMP protein levels. PBMCs from TB-IRIS had increased MMP-1,-3,-7, and-10 transcript levels when compared with those of controls at either 6 or 24 h. Similarly, MMP-1,-3,-7, and-10 protein secretion in stimulated cultures was higher in TB-IRIS than in controls. Serum MMP-7 concentration was elevated in TB-IRIS and 2 weeks of corticosteroid therapy decreased this level, although not significantly. TB-IRIS is associated with a distinct pattern of MMP gene and protein activation. Modulation of dysregulated MMP activity may represent a novel therapeutic approach to alleviate TB-IRIS in HIV-TB patients undergoing treatment.

## Introduction

The introduction of antiretroviral treatment (ART) has been associated with dramatic decreases in mortality and morbidity associated with human immunodeficiency (HIV-1) infection [1]. ART suppresses viral replication, which permits both quantitative and functional reconstitution of the immune system, which is associated with the restoration of pathogen-specific immune responses. While integration of anti-TB and ART therapies is associated with clinical improvement in most patients, HIV-TB associated immune reconstitution inflammatory syndrome (TB-IRIS) may occur in a subset of these patients [2].

TB-IRIS is best recognized as a paradoxical immune-mediated deterioration in patients diagnosed with TB who are responding to anti-TB treatment but develop subsequent clinical deterioration on initiation of ART [2]. The condition has been reported in 8–43% of patients commencing ART while on TB treatment. Mortality due to TB-IRIS averages 3.2%, and morbidity is much more frequent being a major cause of hospitalization [3]. Characteristic features of the condition include swelling and suppuration of lymph nodes, chest x-ray infiltration with cavitation, and formation of tissue abscess [4]. Central to these features is immune-mediated tissue destruction and breakdown of the normal extracellular matrix (ECM) structure.

Matrix metalloproteinases (MMPs) are zinc-dependent endopeptidases capable of degrading all components of the ECM including fibrillar type I collagen, a key structural fibril that is otherwise, highly resistant to enzymatic degradation [5, 6]. The lung matrix is supported by these highly stable fibrillar collagens [7, 8]. MMPs perform multiple roles including tissue remodeling, repair, and modulation of immune responses [9–12]. In healthy tissues, MMPs are rarely expressed: their biological activity is tightly regulated by various mechanisms. Activated MMPs are regulated by endogenous inhibitors, called tissue inhibitors of metalloproteinases (TIMPs) that bind latent and active forms of MMPs [13, 14].

Excessive MMP production has been reported in diverse inflammatory conditions such as cancer, chronic obstructive pulmonary disease, sarcoidosis, interstitial lung disease, arthritis, and atherosclerosis. Advanced pulmonary TB is associated with a locally destructive process of cavitation, which plays an important role in transmission of the disease, but whose pathogenesis remains incompletely understood [10, 15, 16]. Emerging data associates MMP activity with pathology in TB. In recent work, Volkman et al. have demonstrated that disruption of MMP-9 function attenuated granuloma formation and bacterial growth, suggesting that targeting of MMP-9 production could be a promising target for adjunctive immunotherapy [17]. We hypothesized that dysregulated MMP activity may play a critical role in the immunopathology and tissue destruction in TB-IRIS. To investigate this hypothesis, we comprehensively analyzed MMP gene expression and protein secretion in patients who developed paradoxical TB-IRIS and similar non-IRIS control patients.

## Results

### Baseline characteristics of participants

Participants with suspected TB-IRIS were recruited at GF Jooste Hospital, Cape Town, South Africa between March 2005 and December 2006. The 22 TB-IRIS and 22 non-IRIS participants included in the cross-sectional analysis have been previously described [18]. There were no significant differences in the age, gender, and baseline CD4 counts between the two clinical groups. The median time from starting TB treatment, to commencement of combination ART, reporting of TB-IRIS or sample collection was similar in both groups of participants (Supporting Information Table 1).

A second group of participants originated from a randomized placebo-controlled trial (RCT) of prednisone and placebo-treated TB-IRIS patients that has been previously reported [19]. Supporting Information Table 2 shows a summary of the baseline and clinical characteristics for the subset of RCT patients analyzed for the current study. There were no significant differences in gender or baseline CD4 counts between the prednisone-treated (*n* = 16) and placebo-treated (*n* = 12) patient groups. The proportion of participants with previous TB infection, extrapulmonary TB, and IRIS manifestation was similar between the two groups, although a marginal difference (*p* = 0.04) was observed in the median days of TB treatment prior to ART.

### Transcript abundance of MMP genes in TB-IRIS and non-IRIS participants

Relative transcript abundance was assessed by normalizing the cycle threshold (Ct) of the MMP gene of interest with that of the endogenous control, β-Actin. A lower delta Ct value indicates a more abundant transcript and vice versa. Stimulation of PBMC by *Mycobacterium tuberculosis* increased the transcript abundance for multiple MMPs in both the TB-IRIS and non-IRIS groups. At 6 h, MMP-3,-7, and-10 transcripts were significantly more abundant (*p* ≤ 0.05) in the unstimulated controls; after correcting for multiple comparisons, only MMP-3 transcript remained higher (*p*_corr_ = 0.01) in the unstimulated 6 h non-IRIS cultures (results not shown). Upon stimulation with *M. tuberculosis* for 6 h, several of the MMP transcripts including MMP-1, MMP-3, MMP-7, and MMP-10 (*p*_corr_ ≤ 0.05) increased in abundance in both TB-IRIS and non-IRIS patients when compared with those of unstimulated cultures (Supporting Information Table 3).

At 24 h, no significant differences were noted in the transcript abundance of unstimulated IRIS versus non-IRIS PBMC cultures (data not shown). However, after 24 h of stimulation with *M. tuberculosis*, MMP-1, MMP-7, and MMP-10 transcripts were observed to be significantly higher in the PBMC cultures of TB-IRIS as compared with those of non-IRIS patients (*p*_corr_ = 0.02, *p*_corr_ = 0.05 and *p* = 0.01, respectively) (Supporting Information Table 3).

### Fold induction analysis of MMP genes

**Figure 1:**
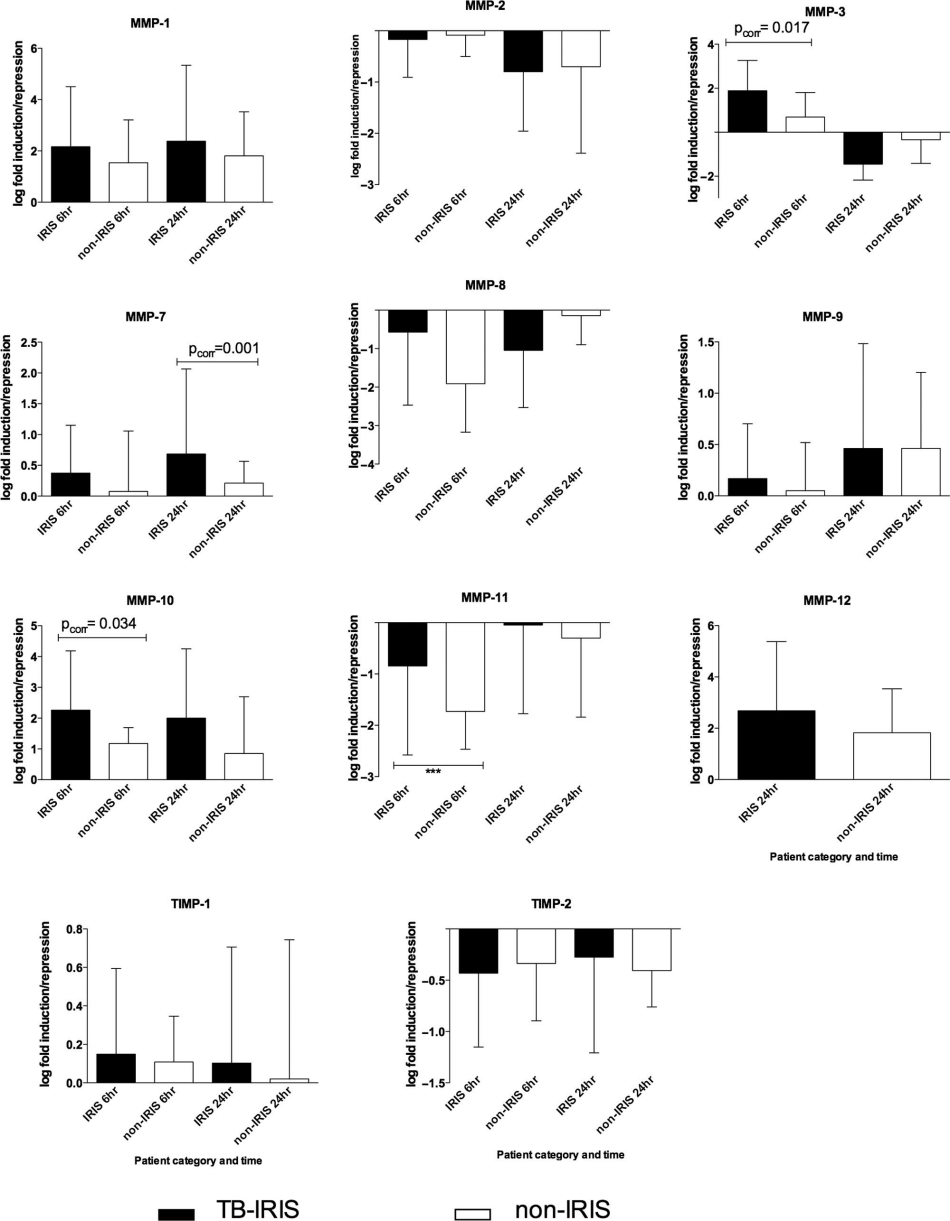
Patients who develop TB-IRIS express increased levels of MMPs. Log-fold induction of MMP genes by heat killed MTB in PBMCs from TB-IRIS at the time of TB-IRIS and non-TB-IRIS control participants who had received a similar duration of antitubercular and antiretroviral therapy. PBMCs from 22 TB-IRIS and 22 controls were cultured in the presence or absence of heat killed H37Rv *M. tuberculosis* for 6 and 24 h. The PBMCs were lysed and mRNA analysis was performed by quantitative RT-PCR. Transcript abundance was calculated by subtracting the Ct β-Actin from the CT of the MMP gene of interest. Fold induction was calculated by the ΔΔCt method and values normalized by Log-10 transformation. Fold induction analysis between IRIS and non-IRIS was performed by unpaired *t*-test. At 6 h, after Bonferroni correction, MMP gene induction was significantly higher in TB-IRIS than in controls for MMP-3, MMP-7, and MMP-10 (*p*_corr_ ≤ 0.05). At 24 h, significant upregulation existed between TB-IRIS and non-IRIS participants for MMP-1, MMP-7, MMP-10, and MMP-12. MMP-12 was undetectable in 6 h cultures. In this figure, the black bars represent TB-IRIS patients while open bars represent non-IRIS controls. Data are shown as median + IQR of 22 TB-IRIS versus 22 non-IRIS control patients for each MMP analyzed (as represented by each panel).

To compare the differences of gene induction between IRIS and non-IRIS, fold induction was determined using the delta delta (ΔΔ) Ct method and values normalized by a Log-10 transformation and analysis performed by an unpaired *t*-test. MMP-1,-3, and-12 tended to be more induced in TB-IRIS at either 6 or 24 h. MMP-7 and MMP-10 were consistently higher at both 6 and 24 h in TB-IRIS patients before correcting for multiple comparisons. After Bonferroni correction, MMP gene induction was significantly higher in TB-IRIS than in non-IRIS controls for MMP-3, and MMP-10 at 6 h (p_corr_ ≤ 0.05), while at 24 h, significant differences existed between TB-IRIS and non-IRIS participants for MMP-7 (*p*_corr_ ≤ 0.001). In contrast, the RNA levels of MMP-2, MMP-8, TIMP-2, and MMP-11 tended to be suppressed by *M. tuberculosis* stimulation at either 6 or 24 h in both groups (Fig. 1).

### MMP protein secretion into PBMC culture supernatants

**Figure 2:**
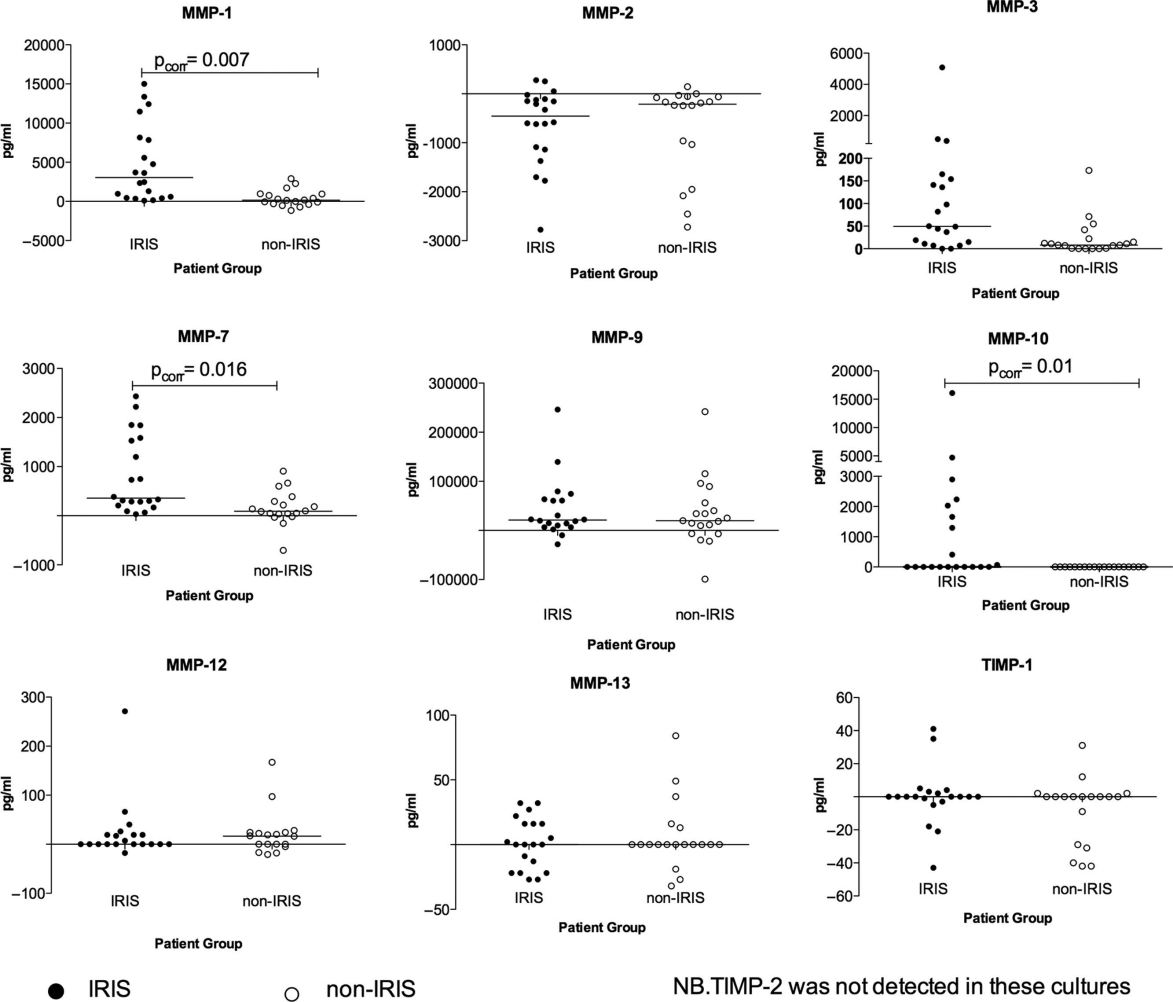
Increased MMP concentrations in PBMC culture supernatants from IRIS patients MMP concentrations were assayed in the PBMC culture supernatants arising from 20 TB-IRIS at the time of TB-IRIS and 20 non-TB-IRIS participants after a similar duration of antitubercular and antiretroviral therapy. The MMP concentrations shown are background subtracted, values being the (delta) difference between stimulated and unstimulated cultures. The delta MMP concentrations of IRIS versus non-IRIS was compared using the Mann–Whitney test. Concentrations of MMP-1,-3,-7, and-10 were found to be significantly higher in the TB-IRIS compared with controls (*p*_corr_ = 0.05). Closed circles represent TB-IRIS patients while open circles represent the non-IRIS controls. Each symbol represents an individual patient and bars represent median values with only significant *p*-values (*p* ≤ 0.0.5) shown on the relevant panels.

MMP protein secretion into the corresponding 24 h PBMC culture supernatants harvested from *M. tuberculosis* stimulated PBMCs was analyzed by luminex and ELISA assays. The MMP concentrations were background subtracted, i.e. the difference between stimulated and unstimulated cultures was calculated and analyzed (Fig. 2). After correction for multiple comparisons, concentrations of MMP-1,-3,-7, and-10 in the PBMC culture supernatants were found to be significantly higher in the TB-IRIS compared with those from controls (*p*_corr_ ≤ 0.05). Although MMP-9 was detected in abundance in these cultures, the levels of this MMP did not differ between the two clinical groups. While TIMP-2 was detected at the transcript level (Supporting Information Table 1), no TIMP-2 protein was detected in these cultures. MMP-9 enzymatic activity on gelatin was determined by zymography (Supporting Information Fig. 1). An abundance of active MMP-9 was observed in both TB-IRIS and non-IRIS groups and this tended to relate well to the MMP-9 levels measured by luminex.

### Correlation of MMP mRNA transcripts

To assess if there were any relationships between different MMP transcripts, we correlated the fold induction of the MMPs with each other. The fold induction values for MMP-1 and MMP-10 transcript were highly and significantly correlated at both 6 and 24 h in both clinical groups (*r* = 0.64–0.89 and *p* ≤ 0.001). Correlation between MMP-1 and MMP-7 was significant only in the IRIS group at both 6 and 24 h (*r* = 0.458 and 0.613, *p* ≤ 0.04). Correlation between MMP-3 and MMP-10 was significant only at 6 h in the IRIS group (*r* = 0.602, *p* = 0.018). No other significant correlations were noted.

### Correlation between MMP transcript and corresponding secreted cell culture supernatant protein

To assess the relationship between MMP transcript and protein secreted into the corresponding supernatant, we correlated the MMP transcript with the 24 h supernatants (Spearman's correlation). As expected, there was an inverse correlation between the 24 h delta CT (mRNA transcript abundance) and secreted protein for MMP-1, MMP-3, and MMP-7. A strong negative correlation was observed between 6 h mRNA (stimulated) and secreted protein for MMP-3 (*r* = −0.626, *p* ≤ 0.0002). No significant correlations were noted for any of the other MMPs.

### Analysis of MMP concentration in serum samples

**Figure 3:**
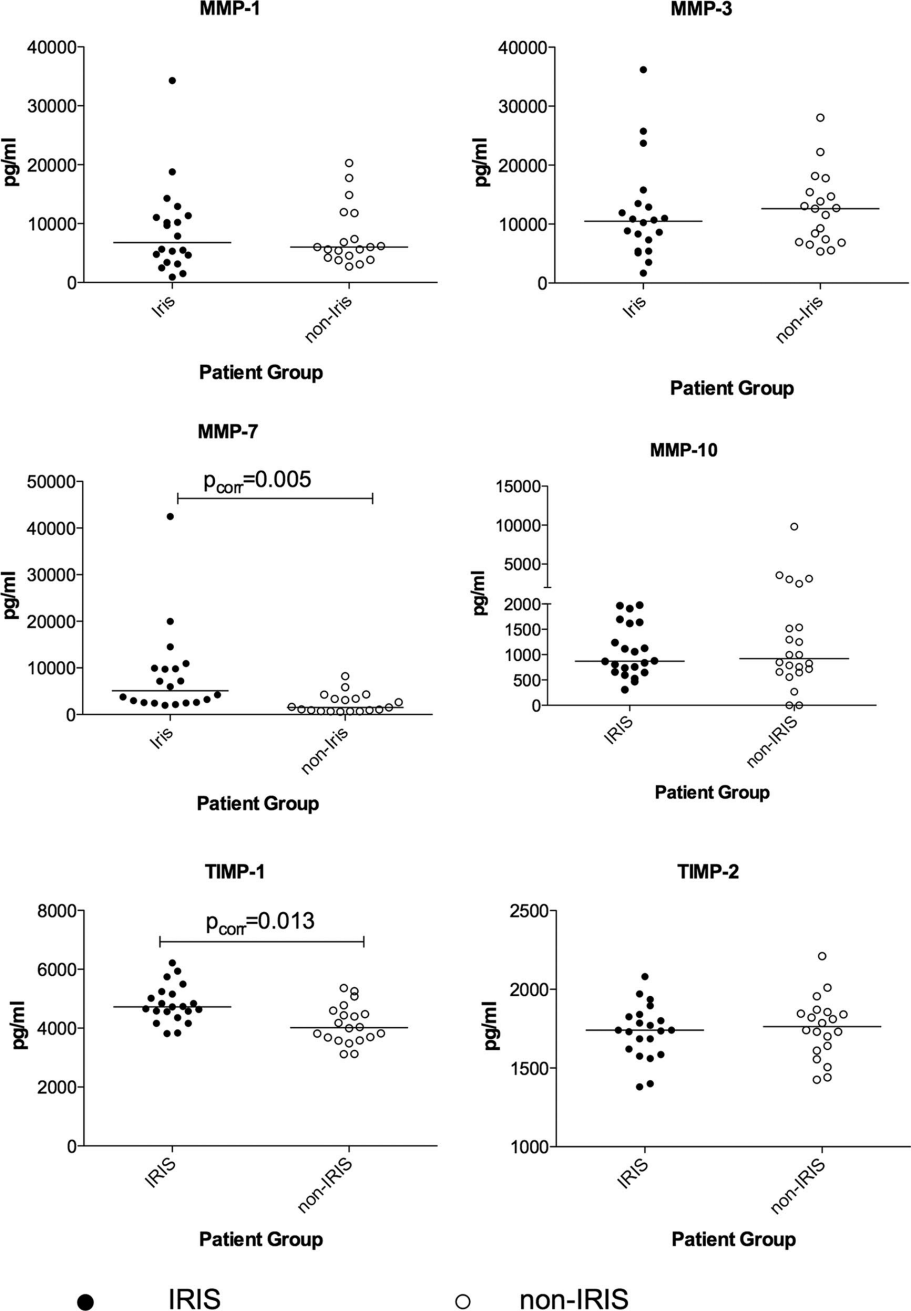
MMP concentrations in the serum of patients. MMP protein levels in the serum samples of 22 TB-IRIS at the time of TB-IRIS and 22 non-IRIS controls who had received a similar duration of antiretroviral therapy were measured by luminex for those analytes shown to be significantly different between IRIS and non-IRIS in the cell culture supernatants. MMP protein levels between IRIS and non-IRIS serum samples were compared using the Mann–Whitney test. MMP-7 protein was significantly higher in serum samples from TB-IRIS when compared with controls (*p*_corr_ = 0.005). The concentration of TIMP-1 protein was also observed to be significantly higher in TB-IRIS (*p*_corr_ = 0.013). In this figure, open circles represent the TB-IRIS patients while the closed circles represent the non-IRIS controls. Each symbol represents an individual patient and the bars represent median values with only statistically significant *p*-values (*p* ≤ 0.05) shown.

To determine whether the increased MMP expression and secretion detected in vitro was reflected in vivo, we analyzed circulating MMPs in corresponding serum samples of the TB-IRIS and control participants. MMP protein levels in the serum of 22 TB-IRIS and 22 controls were measured by luminex for those analytes shown to be significantly different between IRIS and non-IRIS in the cell culture supernatants. MMP-7 was significantly higher in the serum of TB-IRIS compared to controls (*p*_corr_ = 0.005, Fig. 3).

No other significant differences were noted in the concentrations of the circulating proteins between TB-IRIS and control participants for the other MMPs.

### Effect of corticosteroid therapy on gene expression and serum levels in TB-IRIS patients

**Figure 4:**
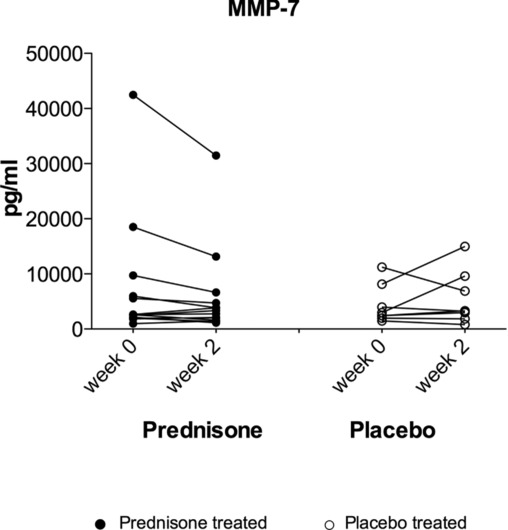
Prednisone tends to suppress circulating MMP concentration in TB-IRIS patients. To assess the effect of steroid therapy on circulating MMP protein concentrations in vivo, (in serum samples) 14 prednisone-treated and 8 placebo-treated TB-IRIS participants were assessed for those MMPs that were higher in IRIS in cell culture supernatants, i.e. MMP-1, MMP-3, and MMP-7. MMP concentrations for each treatment group at the 2-and 4-week time points were analyzed by the Wilcoxon matched-pairs signed rank test. Prednisone tended to suppress MMP-7 concentrations after 2 weeks of therapy although this did not reach statistical significance after correcting for multiple comparisons (*p* = 0.2). In this figure, open circles represent the placebo-treated patients while closed circles represent the steroid (prednisone) treated IRIS patients. Each symbol represents an individual patient.

Prednisone therapy has been shown to improve clinical outcomes in patients presenting with paradoxical TB-IRIS [19]. MMP-1, MMP-3, MMP-7, and MMP-10 gene expression was analyzed by quantitative RT-PCR in *M. tuberculosis* stimulated PBMCs from 16 TB-IRIS participants treated with prednisone therapy compared with 12 patients who were placebo treated over 4 weeks of prednisone versus placebo treatment. Prednisone significantly suppressed MMP-7 gene expression over the treatment course (*p*_corr_ = 0.019). On the other hand, MMP-1 and MMP-3 genes were unaffected by corticosteroid or placebo treatment over the treatment course. Finally, we analyzed circulating MMP concentrations in the serum of TB-IRIS participants over the 4 weeks of treatment. Prednisone tended to suppress circulating MMP-7 concentrations within the first 2 weeks in the prednisone group compared with those after placebo treatment, although this was not significant (*p* = 0.2) (Fig. 4).

## Discussion

We performed a study to investigate the role of tissue degrading MMP enzymes in patients who developed paradoxical TB-IRIS. TB-IRIS is characterized by immune-mediated tissue damage, and therefore MMPs may play a part in this pathology [7, 15, 20, 21]. Our findings show that *M. tuberculosis* stimulation of PBMCs differentially increased the transcript levels for MMP-1, MMP-7, MMP-10, and TIMP-1 genes in paradoxical TB-IRIS participants in 24 h cultures (*p*_corr_ ≤ 0.05) compared with non-IRIS controls. Fold induction was significantly higher in TB-IRIS than in controls for MMP-3 at 6 h, while in the 24 h cultures MMP-1 and MMP-12 were differentially induced in TB-IRIS. MMP-7 and MMP-10 were differentially induced in TB-IRIS at both 6 and 24 h. There was consistency between gene expression and supernatant results with higher MMP protein levels observed in the cell culture supernatants for MMP-1,-3,-7, and-10 that were more highly expressed in TB-IRIS. The upregulation of MMP-1,-3,-7, and-10 by *M. tuberculosis* is consistent with previous reports in primary human macrophages, monocytes, and PBMC [22]. MMP-7 is expressed in macrophages within TB granulomas [15, 22]. Analysis of serum samples showed MMP-7 and TIMP-1 protein levels to be higher in vivo for TB-IRIS compared with those in controls (Fig. 3). Prednisone has been shown to improve clinical outcomes in TB-IRIS patients [19]. By contrast, the transcript levels of MMP-2,-8,-11, and TIMP2 were decreased by restimulation with *M. tuberculosis*. MMP-2 levels in the corresponding cultures were, in many cases, suppressed by the presence of *M. tuberculosis* and TIMP-2 protein was undetectable. These effects did not differ between TB-IRIS and non-IRIS participants and thus we attribute the findings to a general effect of *M. tuberculosis* antigens rather than specific for TB-IRIS.

The involvement of MMPs in lung biology and in tissue degradation in pulmonary TB has been reported previously [5, 20]. However, ours is the first study to investigate the involvement of these tissue-degrading enzymes in paradoxical TB-IRIS. Our findings corroborate related findings on analysis of gene expression and secretion following *M. tuberculosis* stimulation of human microglia in a cellular model of CNS TB [23]. Our findings suggest that MMPs are involved in the immunopathogenesis in TB-IRIS and that infection with MTB promotes a tissue-damaging phenotype that is evident in TB-IRIS [2].

While MMP-9 was highly expressed in the patients we studied, there was no difference in the levels of this MMP in both the mRNA and secreted protein between TB-IRIS and non-IRIS participants. Analysis of MMP-9 enzymatic activity by zymography showed an abundance of active MMP-9 in both TB-IRIS and non-IRIS groups and this tended to relate well to the MMP-9 levels measured by luminex. MMP-9 has previously been implicated in TB immunopathology [9, 24]. More recently, MMP-9 has been implicated in the regulation of monocyte recruitment in granuloma formation and bacterial growth in the *M. marinum* model of infection in zebrafish [17]. We did not demonstrate divergent MMP-9 gene expression or secretion between IRIS and non-IRIS patients in these studies. Thus, while our data support that MMP-9 is associated with the pathogenesis of TB, it does not appear to be specifically associated with the immunopathology that is evident in TB-IRIS.

MMP-7 associated with TB-IRIS and was suppressed by prednisone treatment. MMP-7 may act as a regulator of the immune response in this condition in addition to potentially cleaving fibrils of the ECM [25]. MMP-7 is a multifunctional MMP that is required for activation of defensins [26]. For example, MMP-7 regulates neutrophil egression into the lung by cleaving syndecan-1 to then generate a chemotactic gradient [27] and can also release pro-TNF-α from the cell surface [28]. In other studies, MMP-7 has been shown to regulate the lung localization of dendritic cells to limit inflammation and inhibit fibrosis [29]. MMP-7 activity has also been previously implicated in the pathogenesis of aberrant lung remodeling in pulmonary fibrosis [30].

TIMPs are known to downregulate MMP activity by binding to latent and active forms of MMPs. While TIMPs are constitutively expressed in many tissue fluids, including CSF [31], the peripheral measurement of these inhibitors may not always reflect tissue effects [32]. This may explain our inconsistent findings for TIMPs in this study. TIMP genes were generally not regulated by *M. tuberculosis* at mRNA and cell culture supernatant levels, although TIMP-1 was found to be elevated in the serum of TB-IRIS participants.

We acknowledge some limitations to our study. It is important to note that the mechanisms associated with regulating the potentially destructive nature of MMPs such as binding of active MMPs to their endogenous inhibitors may complicate the quantification of these enzymes in biological samples. Endogenous inhibitors including α-macroglobulin and other TIMPs exert their effect in vivo by trapping active MMPs [33]. Jung and others have demonstrated the importance of blood sample collection for MMP-related analyses and that MMPs and TIMPs concentrations differ between plasma and serum in relation to coagulation and fibrolytic pathways [34, 35]. Anticoagulants have been shown to interfere with MMP activity and so the use of serum may be unreliable for some MMPs. In particular, MMP-1, and MMP-3 are released during blood clotting [34]. Thus, the use of serum as opposed to plasma samples may have obscured some differences in these proteases. The measurement of MMPs in systemic circulation, i.e. in the blood compartment as opposed to the site of disease may only partially reflect production at the site of disease and in the lung [26, 36]. Differences in MMP expression between individual TB-IRIS patients may be attributed to the heterogeneity in the TB-IRIS patient cohort. We acknowledge that our sample size was relatively small and this may have accounted for some borderline significance of some of the reported findings. Further work may be needed to explore these findings in a larger sample size before a clinical trial of MMP inhibitors could be considered in TB-IRIS patients.

**Figure 5:**
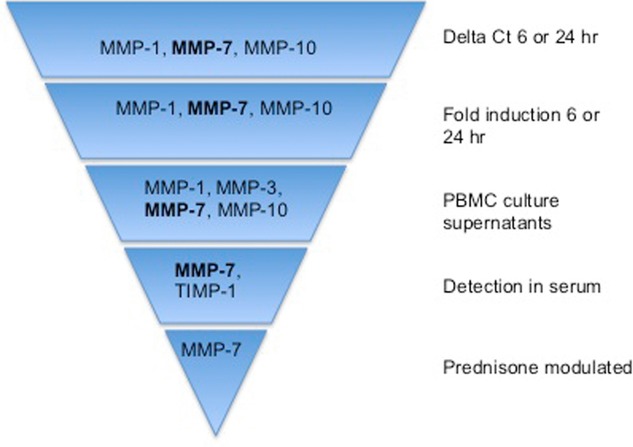
Summary of study findings. While MMP-1, MMP-7, and MMP-10 were consistently elevated in TB-IRIS patients according to experimental circumstances, MMP-7 was the only one that remained elevated in all circumstances, i.e. both in vitro and in vivo. Furthermore, although this did not reach statistical significance, MMP-7 was the only MMP that tended to be modulated by prednisone therapy.

In summary, our studies show that stimulation with *M. tuberculosis* differentially upregulates multiple MMPs in TB-IRIS participants, summarized in Fig. 5. MMP-7 was consistently expressed at increased levels in participants with TB-IRIS and was suppressed by prednisone. As corticosteroids have multiple deleterious immunological effects and may be harmful in the context of drug-resistant TB, more specific immunomodulatory approaches are needed in TB-IRIS. Dysregulated MMP activity may represent a therapeutic target to reduce immunopathology without causing steroid-related immunocompromise. Our findings indicate involvement of MMPs in the immunopathology of paradoxical TB-IRIS, which may present a potential therapeutic target in modulating this condition.

## Materials and methods

### Patient enrolment and study design

Participants were recruited at GF Jooste Hospital and at the Ubuntu Clinic, Site B Khayelitsha, in Cape Town, South Africa between March 2005 and December 2007 to two prospective studies whose designs have been previously reported [19, 37]. TB cases were prescribed 6 months first line therapy of isoniazid, rifampicin, pyrazinamide, and ethambutol (HRZE) for 2 months followed by HR for 4 months (2HRZE/4HR). Patients with previous episodes of TB had streptomycin added to the regimen. All patients were prescribed an ART combination of stavudine (d4T), lamivudine (3TC), and either efavirenz (EFZ) or nevirapine (NVP). Patients suspected or confirmed to have rifampicin or multidrug resistant TB were excluded from the study. Patients were prospectively enrolled into the study and followed up for 12 weeks. Patients were over 18 years of age, not pregnant and ART naive. At entry into these studies, a clinical diagnostic work-up was performed to exclude alternative diagnosis for clinical deterioration. The University of Cape Town Research Ethics committee approved these studies (REC references 337/2004 and 173/2005). All participants provided written informed consent for enrolment into the study.

### Cross-sectional analysis of paradoxical TB-IRIS and non-IRIS patients

For the cross-sectional case-control analysis, TB-IRIS patients were selected from participants who had a confirmed diagnosis of paradoxical TB-IRIS based on the published International Network for the Study of HIV-associated IRIS (INSHI) consensus case definition [2] that has been independently validated [38–40]. The study design, patient selection criteria, and treatment regimens have been previously described [18]. In this analysis, we selected 22 cases of patients (of 32 available) diagnosed with paradoxical TB-IRIS and compared these with 22 non-IRIS control patients who were treated for HIV-associated tuberculosis and had similar baseline CD4 count and duration of ART but did not develop TB-IRIS. We included patients who (i) were ART naive, (ii) had microbiologically confirmed tuberculosis, or conformed to WHO clinical definitions for HIV-associated TB with response to treatment (iii) had no evidence of Rifampicin resistance or MDR, (iv) were diagnosed with paradoxical TB-IRIS based on a validated consensus case definition [2].

### Analysis of randomized placebo-controlled trial (RCT) paradoxical TB-IRIS patients

We have previously described the study design for the RCT of prednisone versus placebo for the management of paradoxical TB-IRIS [19]. Patients presenting with new or recurrent TB symptoms on ART and at least one of the following TB-IRIS manifestations were enrolled: infiltrate on chest radiograph, enlarging lymph node(s), serous effusion, or cold abscess. Exclusion criteria included life-threatening TB-IRIS (mainly neurological involvement). Study medication was prednisone or matching placebo. Participants received study medication for 4 weeks: 1.5 mg/kg per day for 2 weeks followed by 0.75 mg/kg per day for 2 weeks. Blood for MMP analysis was drawn at week 0, 2, and 4.

### Blood processing and cell culture

PBMC were isolated by Ficoll processing of heparinized blood and were plated at 2.5 × 10^6^/well. These were then restimulated with heat killed H37Rv *M. tuberculosis* (prepared in-house from log phase cultures) for 6 or 24 h at a multiplicity of infection of 1:1. After incubation, PBMCs were harvested and lysed for RNA extraction. Cell lysates at 6 and 24 h time points were stored for RNA analysis and the 24 h PBMC culture supernatants were cryopreserved at −80°C. Serum was also collected and cryopreserved at −80°C until further analysis.

### Quantitative RT-PCR

mRNA was extracted from PBMC lysates using the RNeasy Mini Kit Spin Protocol (Qiagen, Valencia, CA, USA) as per manufacturer's recommendations and was cryopreserved at −80°C until further analysis. Primers and probes were purchased from Applied Biosystems as inventoried TaqMan® Gene Expression Assays (Applied Biosystems, Qiagen). The following were the Assay IDs for each of the genes: MMP-1: Hs00899658_m1, MMP-2 Hs00234422, MMP-3: Hs00968308_m1, MMP-7: Hs01042795_m1, MMP-8: Hs01029057_m1, MMP-9: Hs00957555_m1, MMP-10: Hs00233987_m1, MMP-11: Hs00968295_m1; MMP-12: Hs00159178_m1, MMP-13: Hs00233992_m1, TIMP-1: Hs99999139_m1, TIMP-2: Hs00234278_m1. The TaqMan® RNA-Ct 1 Step kit was used in these assays.

Relative transcript abundance was assessed by subtracting the cycle threshold (Ct) of the gene of interest from the cycle threshold of the endogenous control, β-Actin to obtain the difference in thresholds (Δ Ct). A lower delta Ct value indicates more abundant transcript. Fold induction was used to obtain a relative measure of gene induction by MTB and was determined using the ΔΔCt method. ΔΔCt was calculated by subtracting the ΔCt of the unstimulated sample from that of the stimulated sample and adding the resulting value to the mathematical equation 2^−ΔΔCt^ followed to obtain a fold induction/repression of the gene. Values were Log-10 transformed to normalize data before analysis.

### Analysis of PBMC culture supernatant and serum samples

Multiplex assays for MMP were performed on PBMC culture supernatants and serum samples using human MMP multianalyte profiling base kit reagents and Protocol (Human Fluorokine MAP Base Kit, MMP Panel, Cat number LMP000) on the Bio-Rad Bioplex 200 platform. This method detects MMP pro-enzyme content. For MMP-9 analysis, cell culture supernatants were diluted 1:100 before analysis. MMP-10 was measured using the R&D Quantikine ELISA kit (R&D Systems, Cat. number DM1000) which measures human pro-MMP-10 protein with a limit of detection of 4.13 pg/mL. TIMP-1 and TIMP-2 were measured using the R&D Duoset® ELISA Development System (R&D Systems, Cat. numbers: DY970, DY971). For the TIMP-1 and TIMP-2 assays, cell culture supernatant and serum samples were diluted 1:50. For all the cell culture supernatants, the concentrations were background subtracted before analysis and these results are reported.

### Statistical analysis

Data analysis was performed using GraphPad Prism Version 5.0a. The normality of the data was assessed by the D'Agostino & Pearson test. Medians are quoted ± interquartile range (IQR) and means ± SD. Paired parametric data was analyzed by Student's paired *t*-test, or repeated measures one-way analysis of variance (ANOVA). Paired nonparametric variables were analyzed by the Wilcoxon signed rank test or Friedman test. RCT samples were analyzed by Kruskal–Wallis test with no posttest correction. Unpaired parametric variables were assessed using the unpaired *t*-test for parametric data while the Mann–Whitney U test was used for analysis of unpaired nonparametric data. Corrections for multiple comparisons were done using the Bonferroni correction, i.e. *p*-values multiplied by (n-1).

## Abbreviations

ART: antiretroviral treatment
RCT: randomized placebo-controlled trial
TB-IRIS: TB-associated immune reconstitution inflammatory syndrome
TIMP: tissue inhibitors of metalloproteinase

## References

[1] Mocroft A, Vella S, Benfield TL, Chiesi A, Miller V, Gargalianos P, d d'Arminio Monforte A (1998). Changing patterns of mortality across Europe in patients infected with HIV-1. EuroSIDA Study Group. Lancet.

[2] Meintjes G, Lawn SD, Scano F, Maartens G, French MA, Worodria W, Elliott JH (2008). Tuberculosis-associated immune reconstitution inflammatory syndrome: case definitions for use in resource-limited settings. Lancet Infect. Dis.

[3] Muller M, Wandel S, Colebunders R, Attia S, Furrer H, Egger M (2010). Immune reconstitution inflammatory syndrome in patients starting antiretroviral therapy for HIV infection: a systematic review and meta-analysis. Lancet Infect. Dis.

[4] Marais S, Wilkinson RJ, Pepper DJ, Meintjes G (2009). Management of patients with the immune reconstitution inflammatory syndrome. Curr. HIV/AIDS Rep.

[5] Parks WC, Shapiro SD (2001). Matrix metalloproteinases in lung biology. Resp. Res.

[6] Nagase H, Woessner JF (1999). Matrix metalloproteinases. J. Biol. Chem.

[7] Elkington PT, D'Armiento JM, Friedland JS (2011). Tuberculosis immunopathology: the neglected role of extracellular matrix destruction. Sci. Translat. Med.

[8] Elkington PT, O'Kane CM, Friedland JS (2005). The paradox of matrix metalloproteinases in infectious disease. Clin. Exp. Immunol.

[9] Sheen P, O'Kane CM, Chaudhary K, Tovar M, Santillan C, Sosa J, Caviedes L (2009). High MMP-9 activity characterises pleural tuberculosis correlating with granuloma formation. Eur. Respir. J.

[10] Taylor JL, Hattle JM, Dreitz SA, Troudt JM, Izzo LS, Basaraba RJ, Orme IM (2006). A role for matrix metalloproteinase-9 in granuloma formation during pulmonary *Mycobacterium tuberculosis* infection. Infect. Immun.

[11] Gueders MM, Foidart JM, Noel A, Cataldo DD (2006). Matrix metalloproteinases (MMPs) and tissue inhibitors of MMPs in the respiratory tract: potential implications in asthma and other lung diseases. Eur. J. Pharmacol.

[12] Park KJ, Hwang SC, Sheen SS, Oh YJ, Han JH, Lee KB (2005). Expression of matrix metalloproteinase-9 in pleural effusions of tuberculosis and lung cancer. Respiration.

[13] Shapiro SD (1998). Matrix metalloproteinase degradation of extracellular matrix: biological consequences. Curr. Opin. Cell Biol.

[14] Chang JC, Wysocki A, Tchou-Wong KM, Moskowitz N, Zhang Y, Rom WN (1996). Effect of *Mycobacterium tuberculosis* and its components on macrophages and the release of matrix metalloproteinases. Thorax.

[15] Elkington P, Shiomi T, Breen R, Nuttall RK, Ugarte-Gil CA, Walker NF, Saraiva L (2011). MMP-1 drives immunopathology in human tuberculosis and transgenic mice. J. Clin. Invest.

[16] Elkington PT, Emerson JE, Lopez-Pascua LD, O'Kane CM, Horncastle DE, Boyle JJ, Friedland JS (2005). Mycobacterium tuberculosis up-regulates matrix metalloproteinase-1 secretion from human airway epithelial cells via a p38 MAPK switch. J. Immunol.

[17] Volkman HE, Pozos TC, Zheng J, Davis JM, Rawls JF, Ramakrishnan L (2010). Tuberculous granuloma induction via interaction of a bacterial secreted protein with host epithelium. Science.

[18] Tadokera R, Meintjes G, Skolimowska KH, Wilkinson KA, Matthews K, Seldon R, Chegou NN (2011). Hypercytokinaemia accompanies HIV-tuberculosis immune reconstitution inflammatory syndrome. Eur. Resp. J.

[19] Meintjes G, Wilkinson RJ, Morroni C, Pepper DJ, Rebe K, Rangaka MX, Oni T (2010). Randomized placebo-controlled trial of prednisone for paradoxical tuberculosis-associated immune reconstitution inflammatory syndrome. AIDS.

[20] Elkington PT, Friedland JS (2006). Matrix metalloproteinases in destructive pulmonary pathology. Thorax.

[21] Walker NF, Clark SO, Oni T, Andreu N, Tezera L, Singh S, Saraiva L (2012). Doxycycline and HIV infection suppress tuberculosis-induced matrix metalloproteinases. Am. J. Respir. Crit. Care Med.

[22] Elkington PT, Nuttall RK, Boyle JJ, O'Kane CM, Horncastle DE, Edwards DR, Friedland JS (2005). Mycobacterium tuberculosis, but not vaccine BCG, specifically upregulates matrix metalloproteinase-1. Am. J. Respir. Crit. Care Med.

[23] Green JA, Elkington PT, Pennington CJ, Roncaroli F, Dholakia S, Moores RC, Bullen A (2010). Mycobacterium tuberculosis upregulates microglial matrix metalloproteinase-1 and-3 expression and secretion via NF-kappaB-and activator protein-1-dependent monocyte networks. J. Immunol.

[24] Friedland JS, Shaw TC, Price NM, Dayer JM (2002). Differential regulation of MMP-1/9 and TIMP-1 secretion in human monocytic cells in response to *Mycobacterium tuberculosis*. Matrix Biol.

[25] Parks WC, Wilson CL, Lopez-Boado YS (2004). Matrix metalloproteinases as modulators of inflammation and innate immunity. Nat. Rev. Immunol.

[26] Wilson CL, Ouellette AJ, Satchell DP, Ayabe T, Lopez-Boado YS, Stratman JL, Hultgren SJ (1999). Regulation of intestinal alpha-defensin activation by the metalloproteinase matrilysin in innate host defense. Science.

[27] Li Q, Park PW, Wilson CL, Parks WC (2002). Matrilysin shedding of syndecan-1 regulates chemokine mobilization and transepithelial efflux of neutrophils in acute lung injury. Cell.

[28] Haro H, Crawford HC, Fingleton B, Shinomiya K, Spengler DM, Matrisian LM (2000). Matrix metalloproteinase-7-dependent release of tumor necrosis factor-alpha in a model of herniated disc resorption. J. Clin. Invest.

[29] Manicone AM, Huizar I, McGuire JK (2009). Matrilysin (matrix metalloproteinase-7) regulates anti-inflammatory and antifibrotic pulmonary dendritic cells that express CD103 (alpha(E)beta(7)-integrin). Am. J. Path.

[30] Zuo F, Kaminski N, Eugui E, Allard J, Yakhini Z, Ben-Dor A, Lollini L (2002). Gene expression analysis reveals matrilysin as a key regulator of pulmonary fibrosis in mice and humans. Proc. Natl. Acad. Sci. USA.

[31] Rosenberg GA (2002). Matrix metalloproteinases and neuroinflammation in multiple sclerosis. Neuroscientist.

[32] Price P, Mathiot N, Krueger R, Stone S, Keane NM, French MA (2001). Immune dysfunction and immune restoration disease in HIV patients given highly active antiretroviral therapy. J. Clin. Virol.

[33] Visse R, Nagase H (2003). Matrix metalloproteinases and tissue inhibitors of metalloproteinases: structure, function, and biochemistry. Circ. Res.

[34] Mannello F, Jung K, Tonti GA, Canestrari F (2008). Heparin affects matrix metalloproteinases and tissue inhibitors of metalloproteinases circulating in peripheral blood. Clin. Biochem.

[35] Jung K (2008). Matrix metalloproteinase-8 and tissue inhibitor of metalloproteinase-1 in serum do not reflect the analytes circulating in blood. Arterioscler. Thromb. Vasc. Biol.

[36] Murphy G, Nagase H (2011). Localizing matrix metalloproteinase activities in the pericellular environment. FEBS J.

[37] Meintjes G, Wilkinson KA, Rangaka MX, Skolimowska K, van Veen K, Abrahams M, Seldon R (2008). Type 1 helper T cells and FoxP3-positive T cells in HIV-tuberculosis-associated immune reconstitution inflammatory syndrome. Am. J. Respir. Crit. Care Med.

[38] Haddow LJ, Moosa MY, Easterbrook PJ (2010). Validation of a published case definition for tuberculosis-associated immune reconstitution inflammatory syndrome. AIDS.

[39] Eshun-Wilson I, Havers F, Nachega JB, Prozesky HW, Taljaard JJ, Zeier MD, Cotton M (2010). Evaluation of paradoxical TB-associated IRIS with the use of standardized case definitions for resource-limited settings. J. Int. Assoc. Phys. AIDS Care.

[40] Manosuthi W, Van Tieu H, Mankatitham W, Lueangniyomkul A, Ananworanich J, Avihingsanon A, Siangphoe U (2009). Clinical case definition and manifestations of paradoxical tuberculosis-associated immune reconstitution inflammatory syndrome. AIDS.

