# Supplementary material for: Matrix metalloproteinases and tissue damage in HIV-tuberculosis immune reconstitution inflammatory syndrome
Source: Eur J Immunol. 2013 Oct 30;44(1):127–36. doi: 10.1002/eji.201343593 (PMC3992843; doi:10.1002/eji.201343593)
Supplement: Supplementary file 1 [file eji0044-0127-sd1.pdf]

European Journal of Immunology

Supporting Information
for
DOI 10.1002/eji.201343593

Rebecca Tadokera, Graeme A. Meintjes, Katalin A. Wilkinson,
Keira H. Skolimowska, Naomi Walker, Jon S. Friedland, Gary Maartens,
Paul T. G. Elkington and Robert J. Wilkinson

Matrix metalloproteinases and tissue damage in HIV-tuberculosis immune reconstitution inflammatory syndrome

© 2013 The Authors. European Journal of Immunology published by Wiley-VCH Verlag GmbH & Co. KGaA, Weinheim.

Supporting Table 1

Baseline characteristics of TB-IRIS patients and comparator non-IRIS group

	TB-IRIS	Non-IRIS	p-value
n	22	22	NA
Median age (Years, IQR)	31 (23.2 - 52.7)	35.75 (22.2 - 54.1)	0.11
Female n (%)	68	68	0.99
Baseline CD4 /µl, IQR	62 (14.0 -193.0)	42.5 (5.0 – 302.0)	0.17
Median days of TB treatment prior to cART	56 (13.0 -186.0)	75.5 (29.0 -173.0)	0.06
Median days of cART to IRIS or to sample	14 (5.0 -78.0)	14 (14 - 14)	0.94
Previous TB?	8 (36)	3 (14)	0.16
TB disease form n (%)			
Pulmonary or pleural	12 (55)	17 (77)	0.06
Disseminated	7 (32)	4 (18)	0.51
Pericardial	1 (4)	-	
Lymphadenopathic	2 (14)	1 (4)	0.43
Smear or culture confirmed?	19 (86)	13 (59)	0.045

Supporting Table 2: Baseline characteristics of RCT TB-IRIS patients

	Prednisone treated	Placebo treated	p-value
n	16	12	NA
Female n (%)	10 (63)	5 (42)	0.45
Baseline CD4/µL	48<br>[13-181]	68.5<br>[5-156]	0.67
Median days of TB treatment prior to cART	67<br>[22-180]	30<br>[13-115]	0.04
Median days of cART to IRIS onset	14<br>[5-23]	7<br>[3-32]	0.08
Previous TB? (%)	3 (19)	2 (17)	1.00
Basis of TB Diagnosis			
Culture confirmed	9	8	0.71
Smear only	4	2	0.67
Empiric	3	2	1.00
TB Disease Form			
Pulmonary only	9	4	0.28
Extrapulmonary	7	8	0.44
IRIS manifestation n (%)			
New Lymph nodes	6 (38)	3 (25)	0.69
New Abscess	0	0	-
New Pulmonary Infiltrates	3 (19)	3 (25)	1.00
New Effusions	1 (6)	3 (25)	0.29
Recurrent Symptoms	16 (100)	12(100)	1.00
